# Hospital Mortality in Acute Decompensation of Alcoholic Liver Cirrhosis: Can Novel Survival Markers Outperform Traditional Ones?

**DOI:** 10.3390/jcm13206208

**Published:** 2024-10-18

**Authors:** Božidar Dejanović, Otto Barak, Petar Čolović, Nebojša Janjić, Željka Savić, Nikola Gvozdanović, Maja Ružić

**Affiliations:** 1 Faculty of Medicine, University of Novi Sad, 21000 Novi Sad, Serbia; otto.barak@mf.uns.ac.rs (O.B.); nebojsa.janjic@mf.uns.ac.rs (N.J.); zeljka.savic@mf.uns.ac.rs (Ž.S.); 910021d22@mf.uns.ac.rs (N.G.); maja.ruzic@mf.uns.ac.rs (M.R.); 2 Clinic of Gastroenterology and Hepatology, University Clinical Center of Vojvodina, 21000 Novi Sad, Serbia; 3 Department of Psychology, Faculty of Philosophy, University of Novi Sad, 21000 Novi Sad, Serbia; petar.colovic@uns.ac.rs; 4 Clinic of Infectious Disease, University Clinical Center of Vojvodina, 21000 Novi Sad, Serbia

**Keywords:** alcoholic liver cirrhosis, ALC, NLR, FIB4, hospital mortality

## Abstract

**Background**: There is a strong correlation between systemic inflammation intensity and clinical presentation, disease progression, and survival during liver cirrhosis decompensation. This study aimed to evaluate the prognostic performance of blood-based biomarkers as meta-inflammation markers, including NLR, PLR, LMR, INPR, MPR, ALBI, FIB4, and APRI, in predicting hospital mortality in patients with acute decompensation of alcohol-related liver cirrhosis. **Methods**: Data from 411 patients with their first onset of acute decompensation were analyzed, forming two groups: deceased and survived during hospitalization. Generalized partial least squares regression analysis was applied to explore the effects of surrogate indicators on mortality rates, using mortality rate as the dependent variable. Root Mean Square Error, Akaike’s, and Bayesian information criteria determined that four components accounted for most of the variance. **Results**: Variables with significant negative contributions to the outcome prediction (ranked by standardized regression coefficients) were encephalopathy grade, total bilirubin, Child–Turcotte–Pugh score, MELD, NLR, MPV, FIB4, INR, PLR, and ALT. Coefficient sizes ranged from −0.63 to −0.09, with *p*-values from 0 to 0.018. **Conclusions**: NLR, PLR, and FIB4 significantly contribute to hospital mortality prediction in patients with acute decompensation of alcohol-related liver cirrhosis. Conversely, some variables used to predict liver disease severity, including INPR, APRI, LMR, and ALBI score, did not significantly contribute to hospital mortality prediction in this patient population.

## 1. Introduction

Alcohol-related liver cirrhosis (ALC) represents the most advanced and irreversible stage of alcoholic liver disease, stemming from chronic and excessive alcohol consumption. Its prevalence is particularly high in European nations [1,2,3,4].

Global mortality from ALC increased from 676,000 deaths in 1980 (1.54% of global mortality) to 1,029,0420 deaths in 2010 (1.95% of global mortality), while in 2019 it was associated with approximately 2.4% of global deaths [5,6].

Acute decompensation of liver cirrhosis is defined by the appearance of ascites, hepatic encephalopathy and/or variceal bleeding, deriving from acute deterioration of liver function. Ascites is traditionally associated with splanchnic arterial vasodilatation and right ventricular dysfunction, hepatic encephalopathy with hyperammonemia, and variceal bleeding with portal hypertension [7,8,9,10,11].

Classical pathways implicated in progression and decompensation of liver cirrhosis have led to developing scores like the Model for End-Stage Liver Disease (MELD), MELD-Sodium (MELD-Na), Chronic Liver Failure-consortium score for acute decompensated (CLIF-C AD) and Child–Turcotte–Pugh (CTP). Today, they represent well-established and frequently utilized methods for non-invasive assessment of survival in patients with liver cirrhosis. MELD, MELD-Na and CLIF-C ADs scores are difficult to calculate without a personal digital assistant, MELD score, with its wide range of 6–40, which incorrectly predicts mortality in about 15–20% of patients, whereas five parameters are required to interpret and calculate the CTP score, it has a narrow range of disease severity (score range, 7–15) and requires inclusion of subjective criteria such as hepatic encephalopathy and ascites [8,12,13].

Newer research points to the importance of systemic inflammation that acts synergistically with traditional mechanisms involved in the development of liver cirrhosis decompensation. There is a close relationship between the intensity of systemic inflammation and the clinical picture, course and outcome of disease decompensation and patient survival [7,8,9,10,11].

In alcoholic liver disease, the importance of inflammation is emphasized, which has several explanations. Alcohol induces dysbiosis in the gastrointestinal tract, resulting with increased permeability of the intestinal wall (leaky gut), which allows bacterial translocation when endotoxins and alcohol metabolites reach the liver via the enterohepatic circulation, so proinflammatory cytokines and chemokines are consecutively released by complex mechanisms [14]. Furthermore, impaired gastrointestinal motility, portal hypertension, and reduced gastric acid production and secretion are key factors disrupting the gut microbiota, leading to an increase in pathogenic bacteria and a decline in beneficial commensal populations. Through signaling pathways such as the farnesoid X receptor and G protein-coupled receptor 5, the gut microbiota significantly impacts both bile acid composition and liver function [15].

Recently, in addition to standard radiological techniques (abdominal ultrasound, CT, MRI), endoscopic ultrasound (EUS) could play a significant role in the assessment of portal hypertension, as well as in identifying rare pathologies that may contribute to portal hypertension, consequent dysbiosis, and progression to liver cirrhosis. These advanced imaging techniques could provide valuable insights for differential diagnosis, aiding in the comprehensive management of complex liver conditions [16].

In addition, in the reaction with ethanol and its metabolites, free radicals are created, mainly reactive oxygen and nitrogen species (ROS, RNS), and oxidative stress promotes additional tissue damage, stimulating the inflammatory response of the liver, and ultimately fibrosis, cirrhosis and decompensation events [17,18].

Recent progress and understanding of the pathogenesis of liver cirrhosis and acute decompensation, alongside the complexity of existing scoring systems, have underscored the importance and feasibility of identifying survival markers that are easy to obtain, calculate, and interpret.

Inflammatory cells, such as platelets, neutrophils, lymphocytes, monocytes and their ratios Neutrophil-to-Lymphocyte Ratio (NLR), Platelet-to-Lymphocyte Ratio (PLR), Lymphocyte-to-Monocyte Ratio (LMR), International Normalized Ratio-to-Platelet Ratio (INPR), Mean Platelet Volume-to-Platelet Ratio (MPR) [19,20,21,22,23], are proven to correlate with excessive systemic inflammation, crucial for the onset and progression of liver disease. Furthermore, serum markers, such as Fibrosis-4 (FIB4), Albumin–Bilirubin Score (ALBI), and the Aspartate Aminotransferase-to-Platelet Ratio Index (APRI) have demonstrated effectiveness in evaluating liver fibrosis and cirrhosis [24,25,26,27,28].

Our study was focused on evaluating the prognostic performance of various blood-based biomarkers, including NLR, PLR, LMR, INPR, MPR, ALBI, FIB4, APRI in different outcomes of acute decompensation of ALC. We aim to seek out the most significant meta inflammation markers in prediction of hospital mortality among patients with first onset of acute decompensation of ALC, and by so, contribute to further development of noninvasive liver assessment, potentially leading in optimization of clinical decision making and patient management.

## 2. Materials and Methods

### 2.1. Study Population

In this single center retrospective study, we reviewed the data of 411 patients with first onset of acute decompensation of ALC, with etiology based on proven daily intake of pure ethanol, exceeding 30 g for more than 5 years. The patients were consecutively recruited from the Department of Gastroenterology and Hepatology, University Clinical Centre of Vojvodina, Serbia, between May 2019 and May 2024.

This study was conducted in accordance with the Declaration of Helsinki, and the study protocol was approved by the Ethics Committee of the University Clinical Centre of Vojvodina, Serbia (00-98; 19 May 2023).

A diagnosis of liver cirrhosis was based on the patient’s history, clinical examination, laboratory testing, ultrasound and/or computed tomography imaging and upper gastrointestinal endoscopy.

The inclusion criteria were: (a) patients aged over 18 years, and (b) first onset of acute decompensated events due to acute deterioration in liver function, including jaundice, moderate or severe ascites, hepatic encephalopathy or variceal hemorrhage.

Exclusion criteria were: (a) primary liver carcinoma or other malignant tumors with or without metastasis; (b) viral, cholestatic and autoimmune liver disorders; (c) immune-suppressive medications prior hospitalization; (d) ongoing infection (including pneumonia, urinary tract and skin infection); (e) spontaneous bacterial peritonitis; (f) dementia or psychiatric disorders; (g) prior head trauma.

In total, 110 out of 411 patients (26.8%) deceased during the period of hospitalization, due to liver cirrhosis and related complications, such as liver failure, esophagogastric variceal hemorrhage, hepatic encephalopathy (HE), and hepatorenal syndrome (HRS), and were assigned to the deceased cohort group. The rest of the patients, 301/411 (73.2%), survived hospitalization and were discharged with improved clinical condition and laboratory findings, forming the survived cohort group. 

### 2.2. Data Collection

The database included the following: (a) demographic information, including age, sex, duration of alcoholic liver disease; (b) clinical features of liver decompensation; (c) laboratory parameters; upper endoscopy, abdominal ultrasonography and contrast-enhanced computed tomography findings; (d) mortality during hospitalization.

The research material was venous blood samples, and all baseline laboratory tests used in this survey were performed at admission.

**Figure 1:**
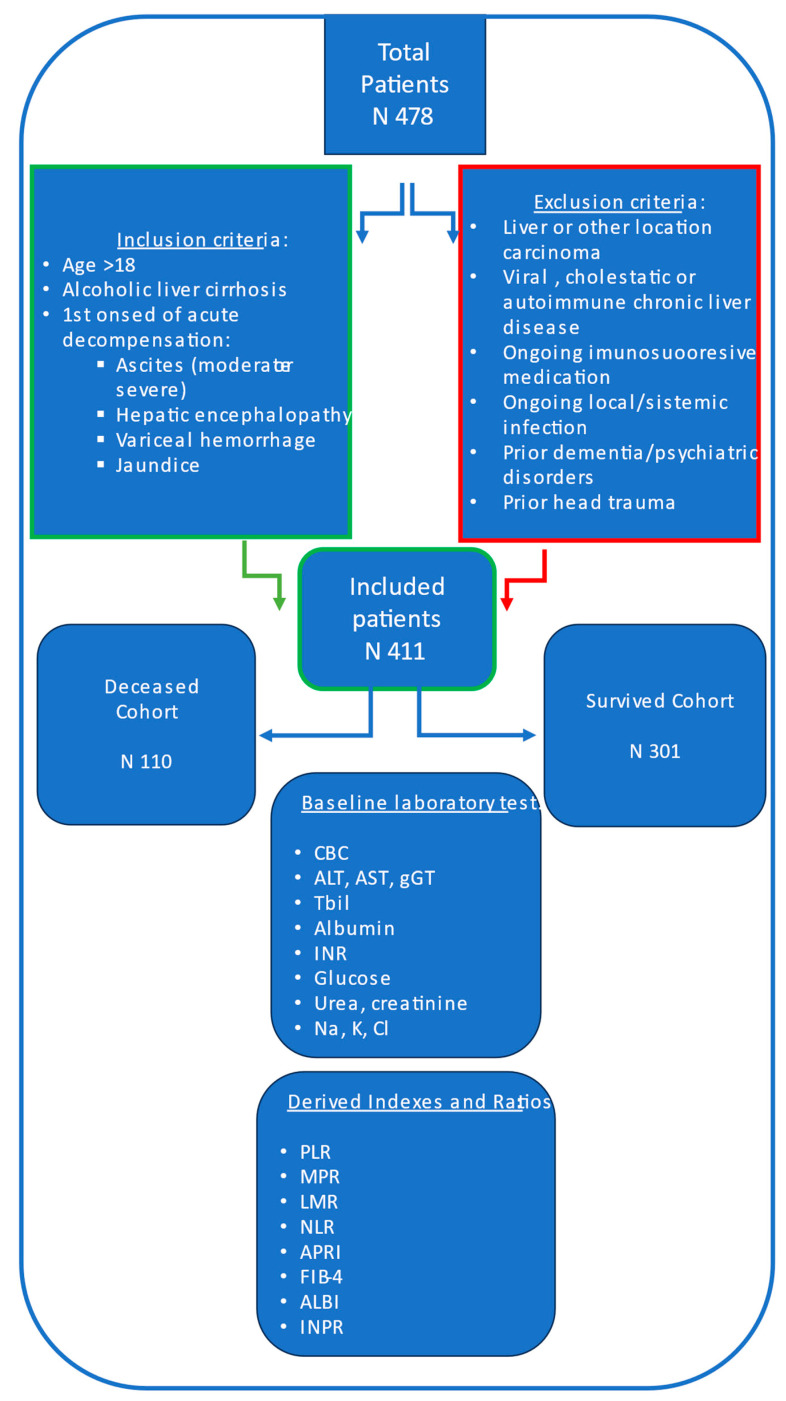
Study flow chart for study population and data collection.

The tested serum indexes, based on complete blood count parameters, including white blood cell, neutrophil (NEU), lymphocyte (LYM), monocyte (MON) and thrombocyte (PLT) count, as well as mean platelet volume and hemoglobin, were: NLR (NEU/LYM), PLR (PLT/LYM), MPR (MPV/PLT), and LMR (LYM/MON). Assessed biochemical parameters were: levels of Alanine Aminotransferase (ALT), Aspartate Aminotransferase (AST), Gamma-Glutamyl Transpeptidase (GGT), Total Bilirubin (Tbil), Albumin, International Normalized Ratio (INR), blood glucose, urea, creatinine, sodium (Na), chlorine (Cl), and potassium (K). Tested biochemical indexes were: APRI (AST/PLT), FIB4 (age × AST/PLT × ALT 1/2), ALBI (−0.085 × Albumin g/L + 0.66 × log Bilirubin [µmol/L]), INPR (INR/PLT × 100). Figure 1 provides a schematic overview of the study population and data collection process.

In addition, CTP and MELD scores were calculated for all patients to evaluate the severity of liver disease.

Abdominal ultrasound was performed in all patients to assess liver echogenicity, presence of focal liver lesion, and ascites. The severity of ascites was ascertained, in accordance with the International Ascites Club, as mild, moderate, or severe on the basis of the ultrasound results: mild ascites (grade 1), radiographic evidence only; moderate ascites (grade 2), manifest by moderate symmetrical distension of the abdomen; and severe ascites (grade 3), with marked abdominal distension and/or paracentesis > 3 L [29].

In this retrospective study, upper endoscopies were performed by endoscopists who simultaneously used two different classifications to define variceal size. The grade I–IV classification (Paquet) was used, as well as the classification of esophageal varices as either small or large, where small varices flatten with insufflation or minimally protrude into the lumen, and large varices protrude into the lumen and touch each other, or they fill at least 50% of the lumen. Variceal bleeding was defined as any chart or procedural documentation implicating varices as the source of gastrointestinal bleeding [30].

### 2.3. Statistical Analysis

To explore the effects of a set of surrogate indicators on patients’ mortality rate, we applied generalized partial least squares (PLS) regression analysis. The dependent (criterion) variable was mortality rate. The set of predictors included are the variables shown in the descriptive statistics in Table 1.

PLS regression methods enable the assessment of predictors’ effects via the latent variables (components) extracted from raw variables’ correlations. In other words, it reduces the set of predictors to a set of linear combinations, each of which comprises mutually correlated predictors whose joint relation with the outcome is substantial. As many components as manifest variables (predictors) can be extracted, usually a smaller number of components accounts for most of the outcome’s variance. Hence, not all components are retained in the analyses, only those that explain substantially more variance than the rest. These “significant” components are chosen according to various criteria, though information measures are most often used [31,32].

Considering the information stated in the previous paragraphs, our estimation of manifest predictors’ contributions is twofold: via the components structure and as standalone predictors. The components’ purposes are to facilitate the potential issues related to the advantages of the PLS estimations over the conventional OLS and logistic (maximum likelihood) estimators for more efficient handling of smaller samples; to highly correlate (even collinear) predictors; and to possibly identify predictor correlations’ patterns and their impact on the outcome [31,33].

PLS methods are helpful in handling large predictor sets by reducing them to smaller numbers of components. In our study, the sample size, although large, did not meet the statistical power-related criteria set for standard logistic regression; hence, we opted for the PLS solution.

**Figure 2:**
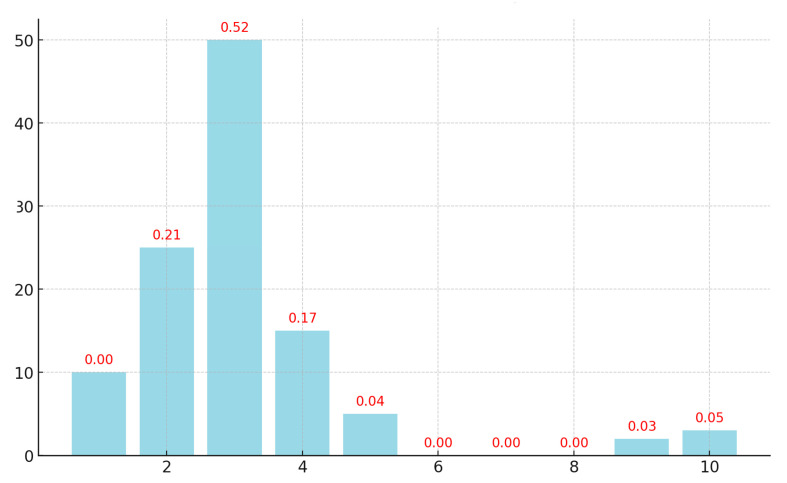
PLS components: variance explained.

We estimated the number of PLS components using RMSE measure, indicating estimation error rates at different component numbers. We tested the solutions from 1 to 10 components. Figure 2 suggests that, after four extracted components, the proportion of variance accounted for did not change substantially. Akaike’s information criterion and the Bayesian information criterion support this result, showing the lowest values at four components.

## 3. Results

Overall, the majority (69.8%) of studied patients were males. The mean age of the population was 59.5 years (SD ± 10.5), and the eldest patient was 88 years old at the moment of hospitalization, while the youngest was 28.

The decompensated events, precipitated by ascites, hepatic encephalopathy and presence of esophageal varices, as well as baseline laboratory findings on admission of both cohorts, are shown in Table 1.

### 3.1. Identification of Derived Predictive Variables on Patient’s Outcome

The number of components to be retained in the analysis was determined using three criteria: the joint proportion of the outcome’s variance accounted for by successively extracted components; Akaike’s information criterion (AIC), an information fit index suggesting smaller values for more plausible component models; and Bayesian information criterion (BIC), suggesting smaller values for better-fitting models. The results suggested the criteria consensus over the optimal number of components, all three suggesting four components as most plausible.

The four components accounted for approximately 90% of the outcome’s variance. Four predictors had salient (over 0.30) loadings on the first component, whereby all loadings were negative: MELD, Child–Turcotte–Pugh score, Tbil, and ALBI. The second component was saliently determined by urea, encephalopathy level, age, creatinine (negative), and monocyte (positive). The third component comprised potassium (positive), FIB4 (negative), and PLR (positive). The fourth component had five variables with salient loadings: white blood cells, platelets, neutrophils (negative), INPRx100, and MPR (positive). Component structure is presented in Table 2.

### 3.2. Effects of Basic Predictive Variables on Patient’s Outcome

The standard errors and *p* values of the predictors’ specific contributions were calculated using a bootstrapping procedure with 1000 samples. The following variables had significant negative contributions to the prediction of the outcome (stated according to the size of their standardized regression coefficients): encephalopathy grade, Tbil, Child–Turcotte–Pugh score, MELD, NLR, urea, age, MPV, FIB4, gender, creatinine, INR, gGT, PLR, and ALT. The coefficient sizes spanned from −0.63 to −0.09, with the respective *p*-values ranging from 0 to 0.018. The variables with positively signed predictor contributions were lymphocyte, MPR, monocyte, Cl, duration of alcoholic liver disease, K, and glucose. Their contributions’ sizes spanned from 0.15 to 0.43, with *p*-values spanning from 0 to 0.028. Predictors’ contributions can be seen in Table 3.

## 4. Discussion

In this study, we demonstrated that various hematological and serum parameters, as well as different ratios, scores and indexes derived from them, have significance in predicting hospital mortality in a population of patients with first onset of acute decompensation ALC.

Peripheral blood platelet and leukocyte count correlated with increased mortality and certain leukocyte subsets and had even greater predictive value in inflammation, tissue damage and overall mortality. Therefore, hematological markers derived from them are seen as good indicators of systematic inflammatory response, allowing us to search for blood-based biomarkers that will serve as simple predictors of hospital mortality in patients with first onset of acute decompensation events of ALC [9,12,34].

NLR has already been proven to have the ability to predict the prognosis of various malignant tumors, cardiovascular diseases and type 2 diabetes mellitus [35,36,37,38]. As an indicator of systemic inflammation, NLR is shedding light on the relationship between two immune pathways. Neutrophil count reflects the presence of ongoing or advancing inflammation, whereas lymphocyte count indicates the activity of immunoregulatory pathways and has notable independent correlation with advanced inflammatory diseases, including liver cirrhosis [22,39].

In our study, NLR showed significant contributions to the prediction of hospital mortality in patients with acute decompensation of ALC, and therefore, showed promising potential. This is in line with the studies by Chiriac et al. [40] and Lin et al. [41], who demonstrated that patients with cirrhosis and a high NLR had higher levels of bilirubin, higher Child–Turcotte–Pugh score and higher incidence of ascites, coagulation, and circulatory failure, presenting a poor outcome.

Rice et al. [42] and Zhang et al. [43] confirmed a nonlinear relationship between the initial NLR and the adjusted likelihood of transplant-free mortality within 90 days, and that a specific NLR range exhibits a strong correlation with unfavorable short-term outcomes in cirrhosis patients. Glisic et al. also showed that NLR is significantly higher in the group of non-survivors compared to the cohort of survivors, with NLR having a prognostic value predicting 30-day mortality in LC [44].

The association between NLR and mortality could be primarily based on the physiological relationship between neutrophilia and lymphopenia, indicative of systemic inflammation and stress, where NLR could signal the patient’s reaction to inflammatory challenges, wherein neutrophil levels rise in response to stress. If stress becomes overwhelming, it may trigger lymphocyte apoptosis. As lymphocytes play a crucial role in regulating an appropriate inflammatory response, their depletion due to apoptosis, cellular exhaustion, and downregulation can perpetuate an adverse inflammatory condition [45,46,47].

Considering MELD [48,49] as a score with high sensitivity, specificity, and positive predictive value to predict 1-month mortality among the patients with decompensated liver cirrhosis, peculiarly, our results confirmed that NLR and MELD score have the exact predictive power (−0.39, *p* = 0), giving us valid reason to consider it as an adequate but much simpler substitute marker of hospital mortality prediction in acute decompensation of ALC.

PLR, as a marker that integrates inflammation and platelet aggregation and crucial events in the occurrence and progression of cardiovascular diseases, has been demonstrated to correlate with the severity and prognosis of conditions such as severe coronary atherosclerosis, myocardial infarction, and heart failure [50,51]. On the other hand, PLR might be used in assessing liver damage, taking into consideration the presence of systemic inflammation, and thrombocytopenia (PLT < 150 × 10^3^/μL), a frequent consequence of chronic liver disease [52].

In the hepatological domain, PLR was primarily investigated in chronic HBV/HCV patients and individuals with HIV/HCV coinfection, typically showing lower PLR values in cases of more advanced liver fibrosis, whereas elevated levels have been noted in patients with hepatocellular disease [44,53,54].

Like NLR, PLR scores among the non-survivors in our study were markedly higher compared to those of the patients who survived, in line with prior investigations carried out by Glisic et al. [44] and Qiang et al. [55]. Yang et al. demonstrated in their work that PLR is associated with the development and fibrosis progression of chronic hepatitis C-related compensated liver cirrhosis [56], probably because both elevated systemic inflammation and thrombocytopenia.

We found PLR to be elevated in patients with acute decompensation of ALC mainly due to systemic inflammation, and consequently, lymphopenia. As thrombocytopenia was present in patients in both of our cohorts, as expected in terminal liver cirrhosis, there was a significant between-group difference. Lymphopenia can be seen in the early stages of cirrhosis and especially in the terminal stadium, affecting both B and T cell populations, including helper and cytotoxic cells. It occurs due to impaired production of new T lymphocytes, accelerated apoptosis, and their depletion resulting from a high load of intestinal antigens. Additionally, it is associated with hindered compensatory proliferation. Some of the aforementioned factors decrease the number of B lymphocytes, consequently leading to heightened susceptibility to bacterial infections [57,58,59]. Long-term alcohol consumption has also been proven to have additional negative influences on the production of antigens and on the population of T cells [60].

MPR, as a hematological parameter, can serve as a prognostic marker in cancer, inflammatory bowel disease, pneumonia and cardiovascular patients. In recent years, its involvement in the progression of liver disorders has also been demonstrated [61].

Cho et al. [62] concluded in their survey that higher MPR was associated with a higher risk of mortality among community-acquired pneumonia patients, while Iida et al. [63] concluded that the MPV/PLT ratio, easily obtained from the complete blood count, could be a simple surrogate marker for predicting LC, since the MPR ratios for patients categorized as fibrosis score F0–1, F2, F3, and F4 were 0.54 ± 0.24, 0.65 ± 0.29, 0.79 ± 0.35, and 1.10 ± 0.51, respectively, with each pairwise difference statistically significant.

In this survey, MPR showed positively signed predictor contributions, with scores markedly lower in the deceased cohort compared to those of the patients who survived. A seemingly unexpected and paradoxical effect of MPR may be provisionally accounted for by its relations to MPV. Namely, MPR is a derivative of MPV, sharing a substantial amount of variance with it. While their bivariate correlations are medium-sized by Cohen’s criteria (Pearson r = 0.317, *p* < 0.001; Spearman rho = 0.466, *p* < 0.001; Kendall’s Tau B = 0.336, *p* < 0.001), one may assume that, due to partialing out the MPV variance in the process of estimating MPR’s effect on the outcome, error (or specific) variance remaining in MPR may have created the signed effect opposite to the expected one. A hierarchical model would be required to confirm the assumption, though the process described above may have been likely due to the mechanics of collinearity and suppression in regression models. One also cannot exclude the possibility of more complex relations among MPV, MPR, and the rest of the predictors, which may have affected the resulting MPR effect.

Fibrosis index based on four factors (FIB4) is traditionally used as a predictor of fibrosis and cirrhosis in patients with hepatitis C and B virus infections and has been validated lately in subjects with non-alcoholic fatty liver disease, while its significance in ALC is yet to be determined. A Japanese study [64] demonstrated that FIB4 could be an effective marker in identifying patients in need for further assessment for alcoholic liver disease, while Roh et al. [65] showed acceptable, both positive and negative, predictive value to diagnose advanced fibrosis in patients with chronic alcoholic liver disease.

Our study revealed that FIB4 significantly contributed to mortality prediction in patients with acute decompensation of ALC, similar to the results of Pan et al. who confirmed that increased FIB4 levels correlate with increased in-hospital mortality, 28-day mortality, and 1-year mortality in critically ill patients with alcohol-use disorder but without alcoholic liver disease [66].

A survey performed on 30 million nonalcoholic fatty liver disease (NAFLD) patients, showed that the FIB4 index was significantly and independently associated with all-cause mortality and liver-related adverse outcomes [67].

After reviewing the literature at our disposal, this is probably the first attempt to examine the predictive significance of this index in hospital mortality among patients with decompensated ALC. While the majority of the patients, in both cohorts, were Child–Turcotte–Pugh B or C class, the platelet count and AST did not have statistically significant differences. In contrast, we found a correlation between age of the patients and ALT with mortality, where we see a possible explanation in emphasizing FIB4 as a predictive marker for hospital mortality in patients with acute decompensation of alcoholic liver cirrhosis.

Some of the variables that are traditionally used in predicting severity of liver disease did not have any significant contributions to the prediction of hospital mortality in patients with acute decompensation of ALC, including platelet count, neutrophile count, serum albumin and AST level. Since most of the patients in both cohorts were, as already stated, CTP B or C class, with high MELD scores, and all with one or more verified signs of acute decompensation of liver cirrhosis, the findings were not unexpected. Therefore, we find the plausible reason for following derived surrogate markers (INPR, APRI, LMR and ALBI scores) not relevant in predicting hospital mortality among patients with acute decompensation of liver cirrhosis, even though they proved to be able to predict the outcome in patients with stable liver cirrhosis [68,69,70,71,72,73,74], and we find it very important to identify this as their prognostic limitations in liver diseases.

## 5. Limitations of the Study and Future Directions

In this survey, we did not analyze the correlation between the dynamic change of tested potential surrogate markers during hospitalization, for example, initial values and 48 h upon hospitalization. Additionally, the study was conducted exclusively on patients with ALC, making it narrowly specific, but at the same time possibly limiting its broader applicability. Given that the study sample was predominantly male (69.8%), this could have introduced potential bias in the results. The significance of gender in acute decompensation of alcoholic liver cirrhosis and its impact on predicting hospital mortality remains to be further investigated.

## 6. Conclusions

This manuscript provides valuable insights into the significance of surrogate markers for predicting mortality in cases of acute decompensation of alcohol-related liver cirrhosis. While we emphasize the importance of identifying reliable indicators to assess the risk of death among these patients, we acknowledge the need for caution in generalizing our findings.

Our study highlights the potential role of NLR, PLR, and FIB4 as valuable tools for predicting hospital mortality, offering easily accessible and cost-effective options to identify patients at higher risk of fatal outcomes. However, it is crucial to approach these results with care and to ensure that timely interventions are based on a comprehensive assessment of each patient.

Conversely, we advise against using INPR, APRI, LMR, and ALBI as surrogate predictor markers for hospital mortality in patients with acute decompensation of alcoholic liver cirrhosis, as our findings suggest limited relevance in this context. Additionally, while MPR demonstrated paradoxical results, further investigation is warranted to clarify its utility in this patient cohort.

In conclusion, our findings contribute to the ongoing discourse in hepatology, while underscoring the importance of continued research to validate these indicators in diverse patient populations and improve clinical decision making.

## Figures and Tables

**Table 1:** (**a**) Baseline Laboratory Findings and Derived Scores and Ratios in Non-Survival and Survival Cohorts. (**b**) Characteristics of Clinical Manifestations in Non-Survival and Survival Cohorts.

(**a**)				
**Baseline Variables**	**Non-Survival** (**Mean Value ± SD**)	**Survival** (**Mean Value ± SD**)	**Welch**	***p* Value**
N	110	301		
White blood cell [×10^9^/L]	10.58 ± 5.81	8.31 ± 4.25	3.748	<0.001 *
Lymphocytes [×10^9^/L]	1.26 ± 0.74	1.62 ± 0.84	−4.166	<0.001 *
Monocytes [×10^9^/L]	0.76 ± 0.55	0.68 ± 0.44	1.597	0.112
Neutrophiles [×10^9^/L]	8.22 ± 4.96	5.86 ± 3.81	4.523	<0.001 *
Platelet [×10^9^/L]	137.19 ± 104.20	150.14 ± 88.38	−1.160	0.248
MPV	10.89 ± 1.09	10.56 ± 1.04	2.718	0.007 *
Erythrocytes [×10^12^/L]	3.23 ± 0.76	3.39 ± 0.71	−1.953	0.052
Hematocrit	0.316 ± 0.067	0.323 ± 0.068	−0.805	0.422
Hemoglobin [g/L]	107.45 ± 23.89	107.56 ± 24.96	−0.043	0.966
MCV	98.0 ± 9.51	95.9 ± 10.39	2.781	0.006 *
Glucose [mmol/L]	6.49 ± 2.21	7.30 ± 2.78	−3.019	0.003 *
Total protein [g/L]	61.39 ± 10.53	64.20 ± 4.47	−2.226	0.028 *
Albumin [g/L]	25.31 ± 4.74	27.61 ± 5.40	−4.178	<0.001 *
Total bilirubin [mg/dL]	175.88 ± 162.34	63.62 ± 68.14	7.000	<0.001 *
AST [IU/L]	141.95 ± 125.26	87.81 ± 89.97	4.144	<0.001 *
ALT [IU/L]	66.80 ± 70.30	49.55 ± 51.28	2.355	0.020 *
gGT [IU/L]	311.96 ± 441.48	232.81 ± 366.56	1.681	0.095
ALP [IU/L]	155.01 ± 164.24	149.27 ± 159.58	0.265	0.791
INR	1.82 ± 0.64	1.43 ± 0.37	5.915	<0.001 *
Na [mmol/L]	134.60 ± 7.39	136.91 ± 5.09	−3.029	0.003 *
K [mmol/L]	4.12 ± 1.01	4.23 ± 8.01	−1.073	0.285
Cl [mmol/L]	97.92 ± 7.46	101.70 ± 5.82	−4.797	<0.001 *
Urea [mmol/L]	14.0 ± 12.92	8.83 ± 7.44	3.967	<0.001 *
Creatinine [μmol/L]	175.16 ± 165.63	112.71 ± 87.66	3.766	<0.001 *
MELD score	25.67 ± 6.98	17.17 ± 6.1	11.205	<0.001 *
CTP score	11.57 ± 1.82	9.48 ± 1.95	10.049	<0.001 *
NLR	8.78 ± 7.24	4.37 ± 3.82	6.065	<0.001 *
LMR	2.24 ± 2.39	2.96 ± 1.85	−2.850	0.005 *
PLR	132.73 ± 118.85	111.83 ± 92.38	1.663	0.098
MPR	0.118 ± 0.08	0.11 ± 0.12	1.138	0.256
INPR×100	2.08 ± 1.65	1.44 ± 1.46	3.544	<0.001 *
APRI	4.98 ± 5.73	2.52 ± 4.24	4.099	<0.001 *
FIB4	11.35 ± 10.02	6.39 ± 6.26	4.840	<0.001 *
ALBI	−0.80 ± 0.54	−1.27 ± 0.56	7.652	<0.001 *
(**b**)				
Clinical manifestation	Non-survival		Survival	
N	110		301	
Encephalopathy	N (%)		N (%)	
None	34 (30.9%)		200 (66.4%)	
Grade 1	23 (20.9%)		34 (11.3%)	
Grade 2	16 (14.5%)		31 (10.3%)	
Grade 3	21 (19.1%)		24 (8.0%)	
Grade 4	16 (14.5%)		12 (4.0%)	
Varices (Paquet classification)				
None	14 (12.7%)		50 (16.6%)	
Grade 1	21 (19.1%)		68 (22.6%)	
Grade 2	23 (20.1%)		92 (30.6%)	
Grade 3	15 (13.6%)		65 (21.6%)	
Grade 4	2 (1.8%)		7 (2.3%)	
No EGD	35 (31.8%)		19 (6.3%)	
Varices (small/large)				
None	14 (12.7%)		50 (16.6%)	
Small	44 (40.0%)		160 (53.2%)	
Large	17 (15.5%)		72 (23.9%)	
No EGD	35 (31.8%)		19 (6.3%)	
Ascites				
None	15 (13.6%)		76 (25.2%)	
Mild	11 (10.0%)		36 (12.0%)	
Moderate/Severe	84 (76.4%)		189 (68.8%)	
CTP Class				
A	0 (0%)		21 (6.9%)	
B	18 (16.4%)		131 (43.6%)	
C	92 (83.6%)		149 (49.5%)	

*—Statistically significant difference (*p* < 0.05); MPV—mean platelet volume; MCV—mean corpuscular volume; AST—aspartate aminotransferase; ALT—alanine transaminase; gGT—gamma-glutamyl transferase; ALP—alkaline phosphatase; INR—international normalized ratio; Na—sodium; K—potassium; Cl—chloride; CTP Class—Child– Turcotte– Pugh Class. MELD—model for end stage liver disease; NLR—neutrophil to lymphocyte ratio; LMR—lymphocyte to monocyte ratio; PLR—platelet to lymphocyte ratio; MPR—mean platelet volume to platelet ratio; INPR×100—international normalized ratio to platelet ratio; APRI—aspartate aminotransferase to platelet ratio index; FIB4—Fibrosis 4 index; ALBI—albumin bilirubin score; EGD—Esophagogastroduodenoscopy.

**Table 2:** Component structure.

	Component 1	Component 2	Component 3	Component 4
MELD	**−0.37**	−0.09	0.09	0.01
CTP_score	**−0.33**	−0.01	−0.06	0.01
Total bilirubin	**−0.30**	0.02	−0.14	−0.04
ALBI	**−0.30**	0.15	−0.03	0.05
INR	−0.25	−0.03	−0.08	0.05
Albumin	0.20	−0.12	−0.08	−0.06
Urea	−0.12	**−0.41**	0.29	0.04
Encephalopathy	−0.14	**−0.39**	−0.08	0.07
Age	0.02	**−0.35**	0.08	0.09
Creatinine	−0.15	**−0.35**	0.23	0.03
Monocytes	−0.13	**0.31**	0.23	−0.19
MCV	−0.14	0.23	−0.13	−0.04
K	0.01	−0.18	**0.36**	0.21
FIB4	−0.20	0.23	**−0.33**	0.24
PLR	−0.05	−0.28	**0.33**	−0.10
APRI	−0.19	0.25	−0.28	0.23
MPV	−0.07	0.01	−0.27	0.09
Na	0.18	−0.11	−0.26	0.10
NLR	−0.23	−0.18	0.24	−0.09
gGT	−0.06	0.13	−0.21	−0.19
Glucose	0.05	0.14	0.19	0.04
AST	−0.17	0.12	−0.18	−0.02
LMR	0.16	−0.11	−0.17	−0.07
ALT	−0.08	0.02	−0.11	0.07
White blood cell	−0.17	0.08	0.27	**−0.38**
INPR×100	−0.16	0.23	−0.25	**0.36**
MPR	−0.10	0.27	−0.24	**0.36**
Platelet	0.04	−0.16	0.32	**−0.33**
Neutrophiles	−0.20	0.02	0.29	**−0.31**
Lymphocytes	0.13	0.18	−0.07	−0.27
Erythrocytes	0.10	−0.20	−0.01	0.25
Duration of ALD	0.03	0.00	0.20	0.23
Cl	0.19	−0.12	−0.09	0.23
Hematocrit	0.04	−0.11	−0.08	0.22
Hemoglobin	0.00	−0.07	−0.09	0.20
Ascites	−0.14	0.11	0.12	−0.14
Gender	−0.06	0.04	−0.02	−0.13

Bolded values indicate predictors that form the structure of each component; ALD—alcoholic liver disease.

**Table 3:** Predictors’ contributions.

	BootstrappedCoefficient	StdCoefficient	BCILower	BCIUpper	Bootp	Bootp.adj
Gender	−0.03	−0.22	−0.04	−0.01	0 *	0.002
Age	−0.04	−0.31	−0.05	−0.01	0.002 *	0.004
Duration of ALD	0.04	0.32	0.01	0.06	0 *	0.002
Ascites	−0.01	−0.04	−0.02	0.01	0.336	0.338
Encephalopathy	−0.07	−0.63	−0.1	−0.04	0 *	0.002
White blood cell	−0.01	−0.11	−0.03	0	0.108	0.110
Lymphocytes	0.02	0.15	0	0.04	0.028 *	0.030
Monocytes	0.03	0.25	0	0.05	0.026 *	0.028
Neutrophiles	0	−0.02	−0.02	0.01	0.604	0.605
Platelet	0	0.01	−0.01	0.01	0.756	0.757
MPV	−0.03	−0.29	−0.05	−0.02	0 *	0.002
Erythrocytes	0.01	0.05	−0.01	0.02	0.388	0.390
Hemoglobin	0.01	0.05	−0.01	0.02	0.34	0.342
Hematocrit	0	0.03	−0.01	0.01	0.642	0.643
MCV	0	0.02	−0.01	0.01	0.782	0.783
Glucose	0.05	0.43	0.02	0.07	0 *	0.002
Albumin	−0.01	−0.05	−0.02	0.01	0.438	0.440
Total bilirubin	−0.06	−0.48	−0.08	−0.04	0 *	0.002
AST	−0.01	−0.04	−0.02	0	0.2	0.202
ALT	−0.01	−0.09	−0.02	−0.01	0 *	0.002
gGT	−0.02	−0.16	−0.03	0	0.002 *	0.004
INR	−0.02	−0.2	−0.04	−0.01	0 *	0.002
Na	−0.02	−0.15	−0.04	0	0.082	0.084
K	0.04	0.37	0	0.08	0.014 *	0.016
Cl	0.03	0.25	0.01	0.05	0.002 *	0.004
Urea	−0.04	−0.34	−0.06	−0.02	0 *	0.002
Creatinine	−0.02	−0.2	−0.04	−0.01	0.006 *	0.008
MELD	−0.05	−0.39	−0.07	−0.02	0 *	0.002
CTP score	−0.06	−0.47	−0.07	−0.04	0 *	0.002
NLR	−0.05	−0.39	−0.06	−0.03	0 *	0.002
LMR	−0.01	−0.07	−0.03	0.01	0.416	0.418
PLR	−0.01	−0.1	−0.02	0	0.018 *	0.020
MPR	0.03	0.23	0	0.05	0.022 *	0.024
INPR×100	0.01	0.07	−0.01	0.02	0.39	0.392
APRI	0	−0.02	−0.02	0.01	0.666	0.667
FIB4	−0.03	−0.26	−0.05	−0.01	0 *	0.002
ALBI	−0.01	−0.08	−0.03	0	0.152	0.154

*—Statistically significant difference (*p* < 0.05); ALD—alcoholic liver disease; BootstrappedCoefficient—regression coefficient obtained by standard bootstrap using 1000 samples with replication; StdCoefficient—standardized regression coefficient; BCILower, BCIUpper—confidence interval, lower and upper margin; Bootp—unadjusted *p* value obtained by bootstrapping procedure; Bootp.adj—adjusted *p* value obtained by bootstrapping procedure.

## Data Availability

The data presented in this study are available on request from the corresponding author.
